# The gender-sensitive spectrum of neurodevelopmental disorders: a case report on a *ZMYM3* variant in a 19-year-old female

**DOI:** 10.3389/fpsyt.2025.1604523

**Published:** 2025-08-15

**Authors:** Alberto Cordella, Silvia Beatrice Maitz, Daniela Distefano, Marianna Lissi, Lucia Morellini, Leonardo Sacco

**Affiliations:** ^1^ Ente Ospedaliero Cantonale, Regional Hospital of Lugano, Department of Neurology, Neurocenter of Southern Switzerland, Lugano, Switzerland; ^2^ Medical Genetics Service, Oncology Department of Southern Switzerland, Ente Ospedaliero Cantonale, Lugano, Switzerland; ^3^ Department of Neuroradiology, Neurocenter of Southern Switzerland, Lugano, Switzerland; ^4^ Neuropsychological and Speech Therapy Unit, Neurocenter of Southern Switzerland, Ente Ospedaliero Cantonale (EOC), Lugano, Switzerland; ^5^ Faculty of Biomedical Sciences, Universitá della Svizzera italiana, Lugano, Switzerland

**Keywords:** neurodevelopmental disorders, *ZMYM3* gene, ADHD, gender differences, genetic testing, neuropsychological assessment, case report

## Abstract

**Background:**

Neurodevelopmental disorders (NDDs) such as Intellectual Disability, Autism Spectrum Disorder (ASD), and Attention-Deficit/Hyperactivity Disorder (ADHD) impact cognitive, behavioral, and social functions. The Zinc finger MYM-type protein 3, located on the X-chromosome, has been implicated in neurodevelopment, but its effects in females remain poorly understood due to limited research.

**Case presentation:**

We report a 19-year-old female with a *de novo* heterozygous variant in *ZMYM3* (NM_201599.3:c.1927C>G, p.(His643Asp)), presenting with ADHD symptoms, poor motor coordination, and mild cognitive impairments. Although her language development was normal, she exhibited motor delays, learning and social difficulties, leading to anxiety and academic struggles. Neuropsychological assessment revealed an IQ of 85, with significant deficits in working memory and visuospatial reasoning but relative strengths in verbal comprehension. Brain MRI showed an incomplete left-sided hippocampal inversion. Genetic analysis confirmed the presence of the *ZMYM3*.

**Discussion and conclusion:**

This case contributes to the limited literature on *ZMYM3*-related NDDs in females, highlighting potential variability in phenotypic expression due to X-inactivation and penetrance effects. The patient’s symptoms emphasize how ADHD and other neurodevelopmental traits may manifest differently in females, often with more subtle and internalized features. Our findings underscore the importance of sex-specific research on *ZMYM3*-associated disorders and the need for comprehensive genetic and neuropsychological assessments to guide diagnosis and intervention in affected individuals.

## Introduction

Neurodevelopmental disorders (NDDs), such as Intellectual Disability, Autism Spectrum Disorder (ASD) and Attention Deficit/Hyperactivity Disorder (ADHD), profoundly affect multiple aspects of life beyond cognitive functioning, beginning from early childhood and often persisting through adulthood. Individuals with NDDs often face challenges in academic attainment, employment, social relationships, and emotional regulation (1). These disorders are also associated with lower self-esteem and increased risks for psychiatric comorbidities, including anxiety and depression, contributing to lifelong impairments in quality of life (1–3).

Often, the impact extends beyond the individual, influencing family dinamics, access to healthcare and inclusion in society, underscoring the importance of early diagnosis, tailored interventions and supportive environments (3).

Zinc finger MYM-type protein 3, located on the X-chromosome, plays a role in neurodevelopment and raises unique issues regarding inheritance and expression (4). Most studies focus on males, creating a gap in understanding its impact in females, where symptoms may be subtler. Hiatt reviewed *ZMYM3*-variants in 27 patients, mostly males, with only three females, highlighting the need for further research (5, 6). This case report discusses a 19-year-old female with a *de novo ZMYM3*-variant, emphasizing her clinical, neuropsychological, and behavioral features.

## Case presentation

**Figure 1:**
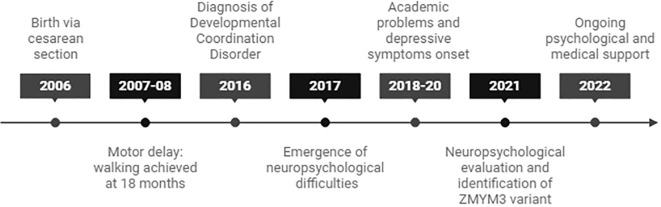
Timeline of key clinical events in the patient’s development.

A 19-year-old female with a family history of neurodevelopmental disorders, including delayed language acquisition in her younger sister and a maternal cousin diagnosed with ASD. A timeline of key clinical events is shown in **Figure 1**.

She was born via emergency cesarean section due to fetal distress and showed mild motor delays, walking at 18 months with hypotonia. Despite motor challenges, her language development was normal. She has bilateral myopia, motor incoordination, and Scheuermann’s disease.

Clinically, she is 176 cm tall with a long face, prominent auricles, narrow palpebral fissures, and a bulbous nasal tip. She has asymmetric pectus carinatum, dorsal kyphosis, and elbow joint hypermobility (Beighton score 2/9). Throughout early schooling in Italy, she struggled with coordination, motor skills, and learning, particularly in math. Diagnosed with developmental coordination disorder at age 10, she later moved to Switzerland, where additional difficulties with attention, reading comprehension, and anxiety emerged. She exhibits symptoms of ADHD, including difficulties focusing and impulsivity, leading to social withdrawal and behavioral outbursts.

Psychologically, she faced bullying and social isolation, which contributed to the onset of depressive episodes. She was described by her parents as socially withdrawn, with increasing signs of anxiety and frustration in academic settings. Academic and social challenges led her to change educational paths, but she eventually discontinued formal education.

Cognitive functions (intelligence, executive functions, attention, visuospatial functions, emotional regulation and behavioral) were assessed. Wechsler Adult Intelligence Scale (WAIS-IV) indicated a heterogeneous cognitive profile. The Verbal Comprehension Index (VCI) was in the average range with a composite score of 96 (39th percentile; 95% CI: 89–103). The Perceptual Reasoning Index (PRI) was in the low average range with a score of 85 (17th percentile; 95% CI: 79–93). The Working Memory Index (WMI) fell in the borderline range with a score of 77 (7th percentile; 95% CI: 72–86). The Processing Speed Index (PSI) was in the average range with a score of 92 (28th percentile; 95% CI: 84–101). The Full Scale IQ (FSIQ) was 85 (15th percentile; 95% CI: 80–91), placing it in the low average range. The General Ability Index (GAI) was 90 (26th percentile; 95% CI: 85–96), and the Cognitive Proficiency Index (CPI) was 82 (12th percentile; 95% CI: 76–90). Both indices were considered interpretable. Due to a significant discrepancy among index scores (range = 19 points), the FSIQ should be interpreted with caution, as it may not reflect a unitary cognitive ability.

Additional neuropsychological assessments showed impairments in executive functions (Five point test and planning in Rey figure copy under 5° percentile, working memory in Digit span backward under 16° percentile), sustained attention (D2-R under 5° percentile), visuospatial processing (Lines orientation under 16° percentile). Emotional regulation and behavioral symptoms were assessed using self-report instruments and informant questionnaires completed by caregivers and teachers (see **Supplementary Data Sheet 1** for details).

**Figure 2:**
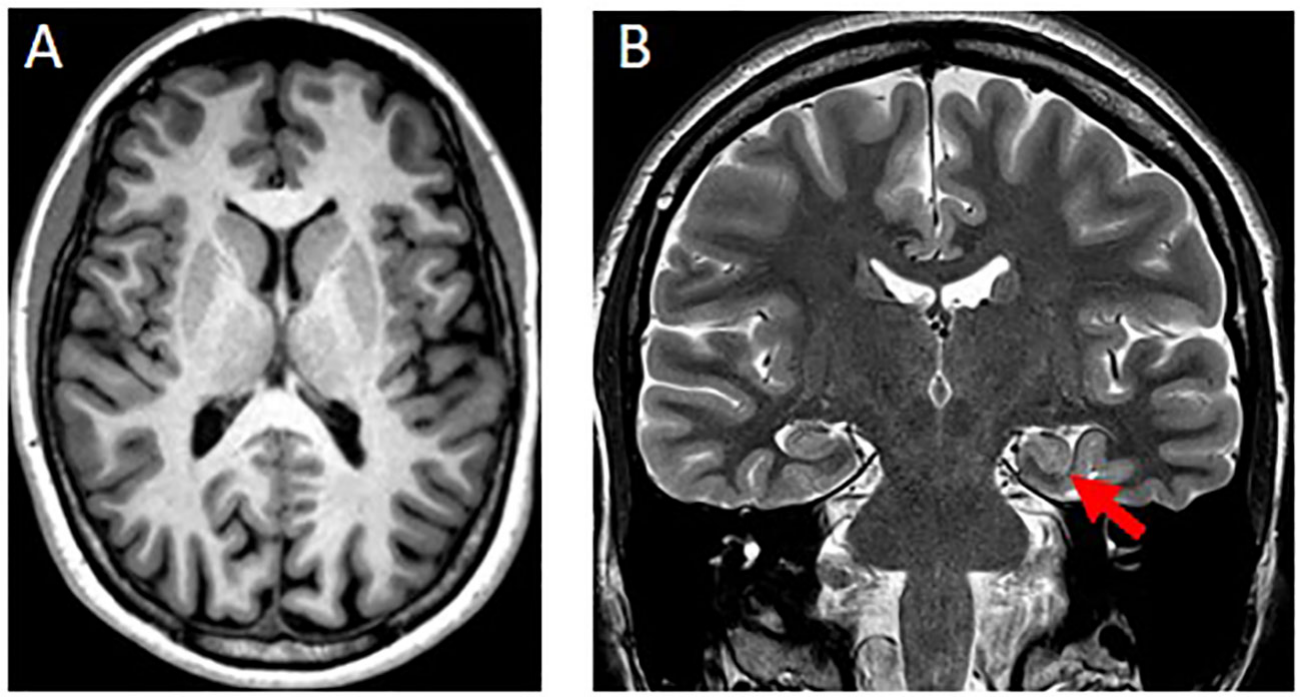
**(A)** Axial T1w-MPRAGE image at the level of the basal ganglia was unremarkable. **(B)** Coronal T2w image showed a round-shaped of the hippocampal head on the left side (red arrow) with normal signal intensity, size and visualization of the internal structure. The left collateral sulcus is more vertical than the controlateral side. These findings are consistent with an incomplete hippocampal inversion.

Brain-MRI showed a left-sided incomplete hippocampal inversion; no other brain *parenchymal* findings were noted (**Figure 2**).

Initial genetic testing for Fragile X and search for CNVs with microarray technology returned negative results, leading to Next Generation Sequencing of over 100 genes in 2021, which revealed a *de novo* heterozygous variant in the *ZMYM3*-gene (NM_201599.3:c.1927C>G, p.(His643Asp)), classified as likely pathogenic according to ACMG criteria: PS2, PM1, PM2.

The ADHD diagnosis was formulated using DSM-5 criteria, applied by experienced clinicians based on all available assessment data (7).

The patient provided informed consent for the publication of this case report.

The patient underwent occupational therapy between ages of 17 and 19, as well as targeted educational support in Switzerland. Behavioral interventions were provided with partial improvement in attentional control and emotional regulation. No pharmacological treatment has been initiated to date.

Actually, the patient is currently not engaged in formal education, but participates in community-based social and occupational support programs. Anxiety symptoms have stabilized, though attentional difficulties and reduced autonomy remain a concern. There have been no hospitalizations or major behavioral problems in the past 12 months. Continued psychological monitoring is ongoing.

In the context of ultra rare disorders, such as those caused by *ZMYM3* variants, we strongly recommend follow-up consultations with a clinical geneticist, as planned for our patient.

The main aim is to reassess the patient’s phenotypic evolution over time, reinterpret genetic findings, provide family counseling and reproductive planning, encourage participation in research and registries, and most importantly, keep the patient and her family informed about scientific and clinical developments relating to the genetic diagnosis.

From the patient’s point of view, the most difficult challenges have been managing attention and emotional regulation, particularly in academic and social environments. Despite this, she preferred not to undertake psychostimulant therapy. She expressed that understanding her condition provided some relief and helped reframe past difficulties. However, uncertainty about future support and outcomes remains a source of anxiety.

“For years, I felt misunderstood in my struggles. When I received the diagnosis and learned about the genetic condition, I felt relieved—it all made sense. It doesn’t fix everything, but now I know it’s not all my fault.”

– Patient, age 19

## Discussion and conclusion

This case provides rare insight into *ZMYM3*-associated neurodevelopmental disorders in females, a group less understood compared to males.

*ZMYM3* encodes a zinc finger MYM-type protein involved in transcriptional regulation and chromatin remodeling. Pathogenic variants are believed to impair its ability to regulate gene expression in the developing brain. This dysregulation may affect genes critical for neuronal migration, synaptogenesis, and cortical connectivity—all essential components of neurodevelopment (5, 13). While *ZMYM3*-variants have been linked to developmental delays and intellectual disabilities in males, their phenotypic expression in females remains underexplored.

In this paper, we seek to highlight the potential role of skewed X-inactivation and dosage effects in the clinical presentation of *ZMYM3*-variants in females, showing variability in symptoms (8, 9).

Our patient presented with ADHD symptoms, cognitive deficit in working memory and visuospatial processing, and emotional challenges such as anxiety and depression. These features align with previously described *ZMYM3*-related phenotypes but also point to potential sex-specific manifestations (10–12). As shown in **Supplementary Data Sheet 2**, we compared the phenotype of our patient with the spectrum observed in previously reported case (5).

Similarities in behavioral and cognitive phenotypes across genetic syndromes, such as Klinefelter syndrome (14, 15), highlight shared pathways underlying NDDs. ADHD-like symptoms, executive dysfunction, and social communication difficulties are common across distinct syndromes, underscoring the complexity and overlap within neurodevelopmental presentations and neurobiological mechanisms (impairment in synaptic development, social cognition or executive function)This case suggests an expansion of the phenotypic spectrum associated with *ZMYM3* variants. It highlights variability that is likely influenced by gene dosage, X-inactivation patterns and the individual’s genetic background. Although functional studies on *ZMYM3* are limited, these observations provide valuable clinical insights. However, definitive causality cannot yet be established without additional functional validation and corroborative evidence. While this case contributes to the understanding of *ZMYM3*-related phenotypes in females, it also raises important questions regarding the underlying molecular mechanisms. In particular, the potential contribution of X-inactivation patterns and dosage sensitivity requires further study. In addition, this case includes a comprehensive neuropsychological profile, which has not previously been reported in female carriers of *ZMYM3* variants.

The main limitation of this report is its single-case design and the absence of functional validation of the identified variant. Although the *de novo* status of the variant, along with the patient’s clinical profile and brain MRI findings, supports a likely pathogenic role, the involvement of additional genetic or environmental modifiers cannot be excluded. Furthermore, no X-inactivation studies were performed in this patient, which prevents us from drawing conclusions about mechanisms such as skewing or allelic dosage imbalance. This is particularly relevant, as skewed X-inactivation is a relatively frequent phenomenon: studies have shown that approximately 25–35% of females in the general population exhibit moderate skewing (>70:30), and 8–10% exhibit extreme skewing (>80:20) in blood-derived DNA (16). Such skewing can influence the expression and penetrance of X-linked variants, as demonstrated in female carriers of Fabry disease or Lesch–Nyhan syndrome, where phenotypic variability has been associated—though not always conclusively—with X-inactivation profiles (17, 18).

In our case, hypotheses involving X-inactivation or gene dosage are raised based on existing literature and the known biology of the gene, but in the absence of direct data they must be interpreted cautiously. Future studies will benefit from incorporating molecular assays such as HUMARA-based methylation testing or RNA-based allelic expression analysis to clarify the relationship between X-inactivation and phenotype expression in female carriers of *ZMYM3* variants.

Therefore, integrated research strategies that combine genomic, epigenetic, and transcriptomic data are necessary to elucidate the mechanisms underlying *ZMYM3*-related disorders in females and to better understand the factors driving phenotypic variability across the sex spectrum.

## Data Availability

The original contributions presented in the study are included in the article/**Supplementary Material**. Further inquiries can be directed to the corresponding author.
