# Supplementary material for: The gender-sensitive spectrum of neurodevelopmental disorders: a case report on a ZMYM3 variant in a 19-year-old female
Source: Front Psychiatry. 2025 Aug 15;16:1604523. doi: 10.3389/fpsyt.2025.1604523 (PMC12394530; doi:10.3389/fpsyt.2025.1604523)
Supplement: Supplementary file 1 [file DataSheet1.pdf]

Scores on psychometric tests and evaluation scales – Regional Civic Hospital of Lugano

Legend: **RS** = rough score, score on test / **CS** = corrected score, obtained by applying correction values necessary to remove the influence on the performance of factors such as age, sex and schooling / **CUT-OFF** = threshold that characterizes a performance as pathological, according to the bibliographical references specific to the instrument/ test and the guidelines of the Swiss Association of Neuropsychologists (ASNP) / **STAT** = statistical reference defined as equivalent score (ES), percentile rank (perc o °), Z value , T value, QI or standard score (SS); if absent, clinical evidence.ES=0 --> ≤ 5°, ES=1 --> >5° and ≤16° ES=2 --> >16° and <35°, ES=3 --> >35° and <50°, ES=4 --> ≥50°/ **LEVEL** = clinical interpretation of performance, marked as poor or limited; * = result to be noted; N.F. = not feasible test.

Neuropsychological evaluation, 08.02.2024

Schooling: 9	***Age: 19***		***Dominant hand: RIGHT***			
	RS		PS	95% IC	STAT	LEVEL
Global cognitive functioning						
Wechsler Adult Intelligence Scale – Fourth Edition.						
Verbal Comprehension Index (VCI)	28		96	-	39°	Medium
Perceptual Reasoning Index (PRI)	23		85	-	17°	Medium-low
Working Memory Index (WMI)	12		77	-	7°	Low
Processing Speed Index (PSI)	17		92	-	28°	Medium
General Ability Index (GAI)	80		85	-	15°	Medium-low
VCI – PRI	96		--	10.81	--	Significant difference
VCI – WMI	96		--	10.75	--	Significant difference
VCI – PSI	96		--	12.71	--	
PRI – WMI	85		--	10.57	--	
PRI – PSI	85		--	12.71	--	
WMI – PSI	77		--	12.66	--	Significant difference
Functional and psycho-behavioral scales						
Barkley Adult ADHD Rating Scales - Self-report: CURRENT SYMPTOMS						
Inattention						
score	17	/36	--	≥93°	83°	
Number of symptoms	2	/9	--	≥93°	89°	
Hyperactivity/impulsivity						
score	23	/36	--	≥93°	95°	Slightly symptomatic
Number of symptoms	5	/9	--	≥93°	99°	Strongly symptomatic

Sluggish Cognitive Time (SCT)						
score	31	/36	--	≥93°	99°	Strongly symptomatic
Number of symptoms	9	/9	--	≥93°	99°	Strongly symptomatic
Total ADHD						
score	40	/72	--	≥93°	94°	Slightly symptomatic
Number of symptoms	7	/18	--	≥93°	96°	Moderately symptomatic
Barkley Adult ADHD Rating Scales - Self-report: CHILDHOOD SYMPTOMS						
Inattention						
score	32	/36	--	≥93°	99°	Strongly symptomatic
Number of symptoms	8	/9	--	≥93°	99°	Strongly symptomatic
Hyperactivity/impulsivity						
score	31	/36	--	≥93°	99°	Strongly symptomatic
Number of symptoms	7	/9	--	≥93°	99°	Strongly symptomatic
Total ADHD						
score	63	/72	--	≥93°	99°	Strongly symptomatic
Number of symptoms	15	/18	--	≥93°	99°	Strongly symptomatic
BARKLEY FUNCTIONAL IMPAIRMENT SCALE-FL						
Mean impairment score	3,78		--	≥93°	91°	Borderline
Percentage of impaired domains	42,86%		--	≥93°	91°	Borderline
Barratt Impulsiveness Scale-11						
total	77	/120	--	>80	--	
Attentional Impulsiveness Factor	21	/32	--	>21	--	Borderline
Attention	13	/20	--	>13	--	Borderline
Cognitive impulsiveness	8	/12	--	>8	--	Borderline
Motor impulsiveness factor	29	/40	--	>29	--	Borderline
motor impulsiveness	19	/28	--	>19	--	Borderline
Perseveration	10	/16	--	>11	--	
Non-Planning Impulsiveness Factor	27	/44	--	>29	--	
Self-control	16	/24	--	>16	--	Borderline
Cognitive complexity	11	/20	--	>13	--	

Mind Wandering						
Mind Wandering – Deliberate (MW-D)	21	/28	--	≥95°	71°	
Mind Wandering – Spontaneous (MW-S)	13	/28	--	≥95°	27°	
Attention Control - Distraction (AC-D)	16	/20	--	≥95°	79°	
AQ – Autism rate	24	/50	--	≥32	--	
DERS- Difficulties in Emotion Regulation Scale						
Total Score	143	/165	--	T≥70	T=102,78	Symptomatic
Non-Acceptance of Emotional Responses	29	/30	--	T≥70	T=88,21	Symptomatic
Difficulties Engaging in Goal-Directed Behavior	25	/25	--	T≥70	T=76,40	Symptomatic
Limited Access to Emotion Regulation Strategies	35	/40	--	T≥70	T=114,99	Symptomatic
Impulse Control Difficulties	30	/30	--	T≥70	T=93,03	Symptomatic
Lack of Emotional Awareness	19	/25	--	T≥70	T=89,11	Symptomatic
Lack of Emotional Clarity	5	/15	--	T≥70	T=47,10	
Attention Control - Shifting (AC-S)	17	/20	--	≥95°	95°	Symptomatic
Mnesic Functions						
Digit Span Forward (Phonological Loop)	5	/9		<4.26	ES=1	Borderline
Digit span Backward	3	/8	2.69	<2.65	ES =1	Borderline
Corsi Block-Tapping Test Forward (visuo-spatial span)	5	/9	4.65	<3.46	ES =2	
Corsi Block-Tapping Test Backward	5	/8	4.28	<3.17	ES =3	
Rey Auditory Verbal Learning Test (RAVLT) – 15-Word List						
Learning	58	/75	49.9	<28.52	ES =4	
Delayed Recall	13	/15	10.2	<4.68	ES =4	
Rey-Osterrieth Complex Figure – Copy	10	/36	4.75	≤28.87	ES =0	*
Attentive Functions						
D2-R of Brickenkamp						
tot correctly crossed out marks (RE)	125		--	≤ 5°	ES =0	*
tot seen marks – mistakes (PC)	99		--	≤ 5°	ES =1	Borderline
% mistakes	20.8		--	≤ 5°	ES =3	

Omissions (Om)	25					
Trail Making Test						
A	44		54	≥127	ES =2	
B	78		91	≥294	ES =4	
B-A	34		37	≥163	ES =4	
B/A	1.77		1.53	≥4.96	ES =4	
Executive Functions						
Five dots test						
Drawings only	28		28.25	<23.84	ES =1	Borderline
Strategies	1		5	<1.90	ES =0	*
Error Index	9.68		--		ES = 4	
Stroop Test						
Time interference effect	16		29.5	≥36.92	ES =2	
Error interference effect	0	/30	0	≥4.24	ES =4	
Visual-spatial and perceptual functions						
Line Orientation	17	/30	17	<17	ES = 1	Borderline
Praxic Functions						
Rey-Osterrieth Complex Figure	28	/36	25.5	≤28.87	ES =0	*
Social Cognition						
Interpersonal Reactivity Index						
Perspective taking	23	/28		≤9.71	ES =4	
Fantasy	17	/28		≤7.78	ES =3	
Personal Distress	13	/28		≤3.54	ES =3	
Empathic Concern	22	/28		≤11.68	ES =4	
Total score IRI	75	/112		≤42.14	ES =4	
