# Supplementary material for: The gender-sensitive spectrum of neurodevelopmental disorders: a case report on a ZMYM3 variant in a 19-year-old female
Source: Front Psychiatry. 2025 Aug 15;16:1604523. doi: 10.3389/fpsyt.2025.1604523 (PMC12394530; doi:10.3389/fpsyt.2025.1604523)
Supplement: Supplementary file 2 [file DataSheet2.pdf]

Reported *ZMYM-3*-associated phenotypes (*Hiatt et al. 2023*) compared with the present patient.

Clinical Feature	Males (n=24)	Females (n=3)	Present case
Global developmental delay (motor)	21/24 (87.5%)	3/3 (100%)	Yes
Speech and language delay	22/24 (91.7%)	3/3 (100%)	No
Intellectual disability (IQ < 85)	18/24 (75.0%)	2/3 (66.7%)	Yes
Autism spectrum disorder or autistic traits	13/24 (54.2%)	1/3 (33.3%)	No
Attention deficit/hyperactivity disorder	9/24 (37.5%)	NA	Yes
Behavioral problems (aggression, self-injury, stereotypies)	12/24 (50.0%)	1/3 (33.3%)	Yes
Dysmorphic craniofacial features	19/24 (79.2%)	3/3 (100%)	Yes
Microcephaly or small occipital frontal circumference (OFC)	8/24 (33.3%)	2/3 (66.7%)	No
Short stature	6/24 (25.0%)	2/3 (66.7%)	No
Feeding or gastrointestinal difficulties	9/24 (37.5%)	2/3 (66.7%)	No
Generalized or axial hypotonia	13/24 (54.2%)	2/3 (66.7%)	No
Visual abnormalities (e.g., strabismus, retinopathy)	5/24 (20.8%)	1/3 (33.3%)	Yes
Hearing impairment	1/24 (4.2%)	0/3 (0%)	No
Epilepsy or seizure-like episodes	2/24 (8.3%)	0/3 (0%)	No
Confirmed de novo ZMYM3 variant	7/24 (29.2%)	2/3 (66.7%)	Yes

Yes: Feature present; **No:** Feature absent; **NA:** Not assessed
